# Risk of Alzheimer's disease or dementia following a cancer diagnosis

**DOI:** 10.1371/journal.pone.0179857

**Published:** 2017-06-20

**Authors:** Erin J. Aiello Bowles, Rod L. Walker, Melissa L. Anderson, Sascha Dublin, Paul K. Crane, Eric B. Larson

**Affiliations:** 1 Kaiser Permanente Washington Health Research Institute, Kaiser Permanente Washington, Seattle, Washington, United States of America; 2 Department of Epidemiology, University of Washington, Seattle, Washington, United States of America; 3 Division of General Internal Medicine, Department of Medicine, University of Washington, Seattle, Washington, United States of America; Istituto Di Ricerche Farmacologiche Mario Negri, ITALY

## Abstract

**Objective:**

We evaluated dementia and Alzheimer’s disease (AD) risks after a cancer diagnosis in a population-based prospective cohort, the Adult Changes in Thought (ACT) study.

**Methods:**

We followed community-dwelling people aged ≥65 years without dementia at study entry for incident dementia and AD from 1994–2015. We linked study data with cancer registry data and categorized cancer diagnoses as prevalent (diagnosed before ACT study enrollment) or incident (diagnosed during follow-up). We used Cox regression to estimate cause-specific hazard ratios (HRs) with 95% confidence intervals (CIs) for dementia or AD risk comparing people with a cancer diagnosis to people without cancer. We conducted sensitivity analyses restricted to people surviving beyond age 80, and stratified by cancer stage, type, and whether the cancer was smoking-related.

**Results:**

Among 4,357 people, 756 (17.4%) had prevalent cancer; 583 (13.4%) developed incident cancer, 1,091 (25.0%) developed dementia, and 877 (20.1%) developed AD over a median 6.4 years (34,482 total person-years) of follow-up. Among complete cases (no missing covariates) with at least one follow-up assessment, adjusted HRs for dementia following prevalent and incident cancer diagnoses were 0.92 (95%CI: 0.76, 1.11) and 0.87 (95%CI: 0.64, 1.04), compared to no cancer history. HRs for AD were 0.95 (95%CI: 0.77, 1.17) for prevalent cancer and 0.73 (95%CI: 0.55, 0.96) for incident cancer. In sensitivity analyses, prevalent late-stage cancers were associated with reduced risks of dementia (HR = 0.51, 95%CI: 0.30, 0.89) and AD (HR = 0.50, 95%CI: 0.27, 0.94). When limited to people who survived beyond age 80, incident cancers were still associated with reduced AD risk (HR = 0.69, 95%CI: 0.51, 0.92).

**Conclusions:**

Our results do not support an inverse association between prevalent cancer diagnoses, which were primarily early-stage, less aggressive cancers, and risk of dementia or AD. A reduced risk of AD following an incident cancer diagnosis is biologically plausible but may reflect selective mortality.

## Introduction

Cancer and Alzheimer’s disease (AD) incidences increase sharply with age; however, epidemiologic data suggest that cancer survivors are less likely to develop AD than people who have never had cancer.[1–6] Disease etiology suggests opposite biologic processes—cancer is defined by uncontrolled tumor cell growth, while AD is associated with neuronal cell death.[7] For example, the protein PIN1, an enzyme involved in protein folding and cell cycle control, can be over-expressed in cancer tumors and under-expressed in brain tissue from people with AD.[8] In addition, p53, a tumor suppressor protein, is inactivated in many cancer cells whereas enhanced p53 activity has been associated with neurodegeneration in people with dementia.[9, 10] Other biological processes involving inflammation and immune function may play a role in this inverse association.[10, 11]

It is possible that inverse associations from observational studies reflect bias from selective mortality. Cancer is the second most common cause of death in the US.[12] People with cancer may appear to have a reduced risk of dementia and AD because of their high mortality risk. People with cancer who die before they have a chance to develop dementia or AD may differ in important ways from those who survive.

Several prior observational studies have noted significant inverse associations between cancer and dementia or AD risk, including a recent meta-analysis.[2–4, 6, 13, 14] The meta-analysis acknowledges limitations of these cohort studies stating that additional “well-designed prospective studies with strict control of confounding factors are needed.”[13] Therefore, we conducted a prospective analysis of the association between cancer diagnoses and dementia and AD outcomes using data from the Adult Changes in Thought (ACT) study cohort adjusting for a wide range of prospectively collected confounders. We evaluated prevalent cancers (diagnosed before study entry when everyone was cognitively health) and incident cancers (diagnosed during study follow-up) separately. We are unaware of any prior study that has evaluated prevalent and incident cancer as separate exposures to see if there is a difference in subsequent dementia or AD risk. We conducted several sensitivity analyses to examine the impact of selective mortality.

## Materials and methods

The ACT study has been described previously.[15–17] In 1994, the ACT study began enrolling community-dwelling adult members of Kaiser Permanente Washington (formerly known as Group Health), a non-profit integrated healthcare system in Washington state, and who lived in or near Seattle. Participants had to be 65 years or older and dementia-free at enrollment. The goal was to conduct a longitudinal study of aging with dementia and AD as primary outcomes; enrollment and follow-up were not related to cancer. ACT enrollment includes: the original cohort enrolled between 1994 and 1996 (n = 2,581), an expansion cohort enrolled between 2000 and 2003 (n = 811), and a continuously enrolled cohort starting in 2004 to maintain a living population of 2000 people. In-person visits occur every two years until the participant dies, develops dementia, or disenrolls from the study. The analyses for this paper include data for 4,357 people collected through August 1, 2015. Analyses were limited to people with at least one follow-up visit because this was the first opportunity for study participants to be diagnosed with dementia or AD. Study participants provided written informed consent and all study procedures were approved by the Institutional Review Board at Kaiser Permanente Washington.

### Exposure

We identified cancer cases by linking ACT study data with the Western Washington Surveillance Epidemiology and End Results (SEER) registry. Kaiser Permanente Washington has linked with SEER since 1974 for primary cancer diagnoses, tumor characteristics, and treatment information within 12 months of diagnosis using methods previously described.[18–20] The Western Washington SEER registry consistently ranks in the top 3 of all SEER registries in terms of data completeness and accuracy,[21] and provides an unbiased source of prospectively collected cancer exposure data as opposed to medical records or self-report. All ACT study participants live within the 13-county area covered by the Western Washington SEER registry. We obtained data on the diagnosis date, stage, site, grade, lymph node involvement, extension of disease, and first course of treatment (including surgery, chemotherapy, radiation therapy, and hormone therapy). We included all cancer types, including reportable skin cancers (excluding basal and squamous cell carcinomas).

We categorized study participants as having a prevalent, incident, or no cancer diagnosis. Prevalent diagnoses occurred any time before ACT study enrollment. Incident diagnoses occurred during ACT study follow-up but before a diagnosis of dementia or AD. We treated cancer as a time-varying exposure. If a person enrolled in the ACT study with no prior cancer diagnosis and then developed a cancer during follow-up, they were counted as having no cancer up until the cancer diagnosis date and counted as having an incident cancer thereafter. If a person had a cancer diagnosis before ACT study enrollment, they were counted as having a prevalent cancer through the end of follow-up unless they developed a second cancer after ACT study enrollment; thereafter, they were counted as having an incident cancer.

### Outcomes

Procedures used to identify incident dementia and AD have been described previously.[15] Briefly, all participants are administered the Cognitive Abilities Screening Instrument[22] every two years. People with screening scores lower than 86/100 receive a full clinical work-up. The final diagnosis is made at a consensus conference based on *Diagnostic and Statistical Manual of Mental Disorders*, *4*^*th*^ *Edition* criteria to define dementia[23] and the National Institute of Neurological and Communicative Disorders and Stroke-Alzheimer’s Disease and Related Disorders Association criteria to define possible or probable AD.[24] We defined the onset dates for dementia and AD as the midpoint between the visit that led to a dementia diagnosis and the previous visit. Deaths from any cause were obtained from Washington State vital statistics and medical record data.

### Covariates

Age, sex, education level, and race were collected via baseline questionnaire. Self-reported hypertension, diabetes, stroke, coronary heart disease, smoking, regular exercise (15 minutes or more at least 3 times per week), and self-rated health were collected at baseline and each follow-up visit. We calculated body mass index (BMI) from height and weight measured at each study visit. *APOE* genotyping has been previously described.[15] We classified participants as *APOE* ε4 positive if they had one or two copies of the ε4 allele.

### Analysis

We summarized baseline and cancer characteristics stratified by baseline cancer status (none or prevalent). We estimated hazard ratios (HRs) and 95% confidence intervals (CIs) of dementia and AD associated with a cancer diagnosis using Cox proportional hazards models with age as the time-scale.[25] Study participants entered the analysis at the age they enrolled in the ACT study. People who had not developed dementia or AD were censored at the age of their last ACT study visit. Models were adjusted for baseline age, ACT cohort, sex, education, and time-varying measures of diabetes, heart disease, stroke, smoking, self-rated health, exercise, and BMI. Some covariates (smoking, self-rated health, exercise, and body mass index) may change because of an incident cancer diagnosis and could be in the causal pathway. We considered adjusting only for baseline values of these variables, but results did not change and we present the models with time-varying covariates. Approximately 13% were missing *APOE* genotype data and adjustment for *APOE* genotype did not substantially change point estimates; therefore, we did not include *APOE* genotype in our final models. Participants with missing covariate data were excluded from the final adjusted models for a complete-case sample size of 4,281. We assessed models for proportional hazards assumption violations using the likelihood ratio test for interactions between exposures and time and visual inspection of residual plots, and found no violations.

We summarized study participants’ follow-up status (still alive, diagnosed with dementia, withdrew, or died) by cancer status at the end of follow-up (no diagnosis, prevalent only and incident cancer). Follow-up status can depend on age at study entry; therefore, we stratified these results by baseline age groups (65–74, 75–84, and ≥85 years).

We conducted sensitivity analyses to evaluate the potential impact of selective mortality. We evaluated whether the risks of dementia and AD differed by cancer stage, hypothesizing that early stage cancers (in situ/ local stage) would be less likely to result in death than late stage cancers (regional/ distant stage). We restricted analyses to people surviving at least to age 80. We limited analyses to more common cancers (breast and prostate) to understand if specific cancer types were associated with dementia or AD risk. We stratified by smoking- and non-smoking-related cancers (smoking-related cancers included oral cavity, pharynx, larynx, esophagus, stomach, pancreas, lung, bladder, or kidney).[2] The reference group for all sensitivity analyses was people with no cancer diagnosis.

## Results

Among 4,357 people in this study, 756 (17.4%) had a prevalent cancer diagnosis before ACT study enrollment and 583 (13.4%) had an incident cancer diagnosis during ACT study follow-up. Participants were followed for a median of 6.4 (IQR 4.0–11.8) years or 34,482 person-years. People with no cancer diagnosis before baseline or a prevalent cancer diagnosis had similar baseline demographic and health characteristics with few exceptions (Table 1).

**Table 1:** Baseline characteristics of ACT study participants by cancer diagnoses before ACT baseline visit. This table shows distributions of baseline demographic and health characteristics of the ACT study population, stratified by whether a person was diagnosed with any prevalent cancer at baseline. Abbreviations include: ACT (Adult Changes in Thought); IQR (interquartile range); and BMI (body mass index).

Baseline variable	No cancer diagnosis before ACT baseline visit (n = 3601)		Prevalent cancer diagnosis before ACT baseline visit (n = 756)	
	N	%^1^	N	%^1^
Age (median, IQR)	73	(69–78)	75	(70–80)
Follow-up yrs (median, IQR)	7.0	(4.0–11.9)	6.0	(3.9–9.9)
ACT cohort: Original	1933	(53.7)	366	(48.4)
Expansion	601	(16.7)	136	(18.0)
Replacement	1067	(29.6)	254	(33.6)
Sex: Female	2109	(58.6)	450	(59.5)
Male	1492	(41.4)	306	(40.5)
Education: < College degree	1770	(49.2)	371	(49.1)
College degree or more	1829	(50.8)	385	(50.9)
Race: White	3204	(89.0)	694	(91.9)
Black	148	(4.1)	14	(1.9)
Asian	134	(3.7)	20	(2.6)
Other	112	(3.1)	27	(3.5)
Ethnicity: Not Hispanic	3559	(99.0)	749	(99.2)
Hispanic	37	(1.0)	6	(0.8)
Diabetes: No	3215	(89.6)	662	(87.7)
Yes	375	(10.4)	93	(12.3)
Hypertension: No	2096	(58.8)	446	(59.2)
Current and treated	1290	(36.2)	272	(36.1)
Current untreated	179	(5.0)	35	(4.6)
Heart Disease^2^: No	2954	(82.6)	619	(82.5)
Yes	622	(17.4)	131	(17.5)
Stroke: No	3492	(97.3)	724	(96.0)
Yes	97	(2.7)	30	(4.0)
Smoking: Never smoker	1763	(49.1)	350	(46.4)
Past smoker	1660	(46.2)	364	(48.3)
Current smoker	171	(4.8)	40	(5.3)
Low self-rated health: No	3104	(86.5)	618	(81.7)
Yes	486	(13.5)	138	(18.3)
BMI: <25	1174	(33.2)	233	(32.1)
≥25-<30	1438	(40.7)	301	(41.5)
> = 30	920	(26.0)	192	(26.4)
Exercise regularly: No	999	(27.8)	242	(32.1)
Yes	2591	(72.2)	513	(67.9)

^1^% among non-missing; numbers may not add to totals due to missing data.

^2^Includes any self-reported myocardial infarction, angina, coronary artery bypass grafting, or angioplasty

A greater proportion of prevalent cancers were diagnosed at younger ages and early stages or low grades compared with incident cancers (Table 2). This may be driven by our study design because prevalent cancers had to be diagnosed before ACT study baseline, and people with more advanced cancers in the past may have had less chance of surviving to age 65 and ultimately enrolling in ACT. Breast and prostate cancers accounted for 46.9% of prevalent cancer diagnoses and 37.9% of incident cancer diagnoses. Greater proportions of people with incident cancers were treated with chemotherapy than people with prevalent cancers; however, equal proportions of both groups received radiation therapy.

**Table 2:** Characteristics of cancers diagnosed before baseline visit and during follow-up in ACT study participants. This table shows the distributions of cancer characteristics among people diagnosed with a prevalent or incident cancer. Abbreviations include: ACT (Adult Changes in Thought) and IQR (interquartile range).

Cancer characteristic	Prevalent cancer diagnosis before ACT baseline visit^1^ (n = 756)		Incident cancer diagnosis during ACT follow-up^1^ (n = 583)	
	N	%^2^	N	%^2^
Age at diagnosis (median, IQR)	67.4	(61.3–73.0)	79.1	(74.7–84.6)
Years between ACT baseline visit and diagnosis date (median, IQR)^3^	-7.2	(-13.0–-3.4)	4.9	(2.4–8.3)
Year of diagnosis: 1974–79	69	(9.1)	0	0
1980–89	230	(30.4)	0	0
1990–99	308	(40.7)	149	(25.6)
2000–09	136	(18.0)	286	(49.1)
2010–14	13	(1.7)	148	(25.4)
Summary stage: in situ	107	(14.5)	75	(13.4)
local	476	(64.3)	303	(54.3)
regional	134	(18.1)	86	(15.4)
distant	23	(3.1)	94	(16.8)
Grade: well differentiated	106	(14.0)	55	(9.4)
moderately differentiated	261	(34.5)	174	(29.8)
poorly differentiated	90	(11.9)	98	(16.8)
undifferentiated	13	(1.7)	32	(5.5)
Cancer site: oral cavity/pharynx	19	(2.5)	14	(2.4)
colon and rectum	109	(14.4)	56	(9.6)
other digestive system	11	(1.5)	11	(1.9)
lung and bronchus	8	(1.1)	32	(5.5)
soft tissue including heart	0	0.0	6	(1.0)
skin	62	(8.2)	59	(10.1)
breast	215	(28.4)	119	(20.4)
female genital system	85	(11.2)	28	(4.8)
prostate	140	(18.5)	102	(17.5)
urinary system	54	(7.1)	58	(10.0)
lymphoma	32	(4.2)	48	(8.2)
Chemotherapy: No/unknown	678	(89.7)	500	(85.8)
Yes	78	(10.3)	83	(14.2)
Radiation therapy: No/unknown	517	(68.4)	417	(71.5)
Yes	239	(31.6)	166	(28.5)

^**1**^Prevalent and incident cancers are not mutually exclusive; 107 people had a prevalent cancer diagnosis before baseline and an incident diagnosis during follow-up and are included in both columns.

^2^% among non-missing; numbers may not add to totals due to missing data and exclusion of small cell sizes (<5)

^3^Median time between ACT baseline visit and diagnosis date is negative for prevalent cancers because they were diagnosed before the ACT baseline visit and positive for incident cancers because they were diagnosed after the ACT baseline visit.

During the study period, 1,091 people were diagnosed with dementia including 877 possible/probable cases of AD (Table 3). The adjusted HRs for dementia were 0.92 (95%CI: 0.76, 1.11) for prevalent cancer diagnoses and 0.82 (95%CI: 0.64, 1.04) for incident cancer diagnoses. When evaluating AD risk, the adjusted HR was only statistically significant for incident cancers (HR = 0.73, 95%CI: 0.55, 0.96).

**Table 3:** Risks of dementia and AD after a cancer diagnosis among ACT study participants. This table shows follow-up time, dementia and AD incidence, and crude and unadjusted risks of dementia and AD for prevalent and incident cancers. Abbreviations include: ACT (Adult Changes in Thought); AD (Alzheimer’s disease); CI (confidence interval); and HR (hazard ratio).

Dementia	follow-up time(person-years)	# events	Incidence per 1000 person years	95% CI	Crude HR^1^	95% CI	Adjusted HR^2^	95%CI
No cancer	26,735	839	31.4	29.3, 33.6	1		1	
Prevalent cancer	4,872	154	31.6	27.0, 37.0	0.93	0.78, 1.10	0.92	0.76, 1.11
Incident cancer	2,874	98	34.1	28.0, 41.6	0.82	0.66, 1.01	0.82	0.64, 1.04
**Possible/Probable AD**								
No cancer	26,735	678	25.4	23.5, 27.3	1		1	
Prevalent cancer	4,872	126	25.8	21.7, 30.8	0.93	0.77, 1.13	0.95	0.77, 1.17
Incident cancer	2,874	73	25.4	20.2, 31.9	0.73	0.58, 0.94	0.73	0.55, 0.96

^1^Crude HR uses age as the time scale

^2^Additionally adjusted for age at ACT study entry, ACT cohort, gender, education, diabetes, hypertension, heart disease, stroke, smoking status, low self-rated health, regular exercise, and body mass index

Table 4 summarizes follow-up status by baseline age groups and final cancer status. People with incident cancers had the lowest proportions of study withdrawals (4.0–5.5%) and the highest proportions of deaths (35.8–60.0%) within each age group.

**Table 4:** ACT follow-up status by age at enrollment in the ACT study and final cancer exposure group. This table shows the proportion of people by ACT study follow-up status (still alive, diagnosed with dementia, withdrew from the ACT study, or died) stratified by baseline age groups and cancer exposure group. Abbreviations include: ACT (Adult Changes in Thought).

Age/cancer group	N	ACT study status at the end of follow-up			
		Still alive in study	Dementia diagnosis	Withdrew from study	Died
Baseline age 65–74		Row %	Row %	Row %	Row %
No cancer	1818	53.0	18.5	7.6	20.9
Prevalent cancer	311	48.2	13.5	10.9	27.3
Incident cancer	377	44.3	15.4	4.5	35.8
Baseline age 75–84					
No cancer	1068	21.0	37.3	10.5	31.3
Prevalent cancer	267	19.1	34.5	9.0	37.5
Incident cancer	181	19.9	18.2	5.5	56.4
Baseline age 85+					
No cancer	239	8.4	43.5	8.0	40.2
Prevalent cancer	71	15.5	28.2	12.7	43.7
Incident cancer	25	8.0	28.0	4.0	60.0

In sensitivity analyses, prevalent late-stage cancers (S1 Table) were associated with reduced risks of dementia (HR = 0.51, 95%CI: 0.30, 0.89) and AD (HR = 0.50, 95%CI: 0.27, 0.94). When limiting to 2,787 people who survived at least to age 80, incident cancers were associated with a reduced risk of AD (HR = 0.69, 95%CI: 0.51, 0.92, S2 Table). There were no statistically significant associations between breast or prostate cancers (S3 Table) or smoking-related cancers (S4 Table) and dementia or AD.

## Discussion

Our results were similar to those from prior observational studies and one meta-analysis, in that having cancer was associated with a decreased risk of AD (as measured by the cause-specific hazard), but we only observed this for incident cancers. People with prevalent cancers did not have a significantly lower risk of dementia or AD (HRs = 0.92 and 0.95, respectively); however, people with incident cancers had a non-significant reduced risk of dementia (HR = 0.82) and a significantly reduced risk of AD (HR = 0.73) compared to people without cancer. This finding may be related to a greater mortality risk among people with incident cancers, but does not preclude a biologic explanation.

There is potential for informative censoring in our study from some unmeasurable factor that is associated with a different mortality risk in one group than another. If people with an incident cancer died before they had a chance to be diagnosed with dementia or AD and they were systematically different in unmeasured ways from people with an incident cancer who were diagnosed with dementia or AD, any inverse association between incident cancers and dementia or AD could be due to selective mortality. People diagnosed with incident cancers may have had a poor cancer prognosis as evidenced by late stages, high tumor grades, and a substantial proportion of deaths. If incident cancers were more aggressive than prevalent cancers in this cohort, they may have been associated with greater PIN1, less p53 expression, or changes in inflammation or immune function—any of which could reduce dementia risk. Therefore, while there is a possibility of bias due to informative censoring, there exists a plausible biologic rationale for a reduced risk of AD following incident cancer.

People with a prevalent cancer diagnosed before ACT study entry presumably had been treated and recovered from their cancer because they were healthy enough to join a study unrelated to their cancer diagnosis. By the time they entered the ACT study, their prior cancer diagnosis may not have affected their future mortality risk compared with similar aged people in ACT without prior cancer. However, individuals with a prevalent diagnosis might be more robust, possibly in unmeasured ways, and thus not representative of all people with cancer. For example, it is possible that people with aggressive cancers prior to ACT enrollment were too sick to enroll in the study. On average, participants with prevalent cancer had tumors that were less aggressive than incident cancers. Less aggressive tumor biology among that group may explain why we found no association between prevalent cancers and dementia or AD. This was the case in every sensitivity analysis with one exception—people diagnosed with a prevalent *late-stage* cancer did have significantly reduced risks of dementia and AD. Late stage cancers may have had a more aggressive biology with greater expression of PIN1 or underexpression of p53 –supporting a biological explanation for a reduced risk of dementia and AD.

Several previous studies and a meta-analysis found inverse associations between any cancer diagnosis and dementia or AD risk.[2, 4, 13, 14] However, our results showed that incident and prevalent cancers should not be combined into a single “any cancer” group because they have different associations AD and dementia. We are only aware of two prior studies that limited analyses to prevalent cancers. Both studies found inverse associations between a prevalent cancer diagnosis and AD; however, neither one provided information on cancer stage or aggressiveness.[5, 6] The Cardiovascular Health Study-Cognition Substudy[6] showed an inverse association between prevalent cancer and any AD: HR = 0.72 (95%CI: 0.52, 0.997) among 2,151 participants with no dementia at baseline (age 65, mean 5.4 years of follow-up). An Italian case-control study[5] with 126 probable AD cases and 252 matched controls showed that a prior cancer diagnosis (on average 16–17 years prior to AD onset and ascertained via a caregiver questionnaire after AD diagnosis) was associated with a reduced odds of AD (OR = 0.6, 95%CI: 0.4, 1.1). The self-reported and administrative claims data used in these studies are reasonable for classifying exposure and outcome data; however, they may be less valid than cancer data from a tumor registry or consensus-based dementia and AD diagnoses from a full neuropsychological and clinical evaluation. Additional differences in methods, including confounder adjustment, may explain different results between our study and these reports.

One important limitation of our study was we could not evaluate associations between cancer treatment and dementia or AD because SEER did not have detailed treatment data for all study years. In addition, our SEER registry started in 1974 and only covers diagnoses in 13 counties in western Washington state. We may be missing diagnoses that occurred prior to 1974 or outside of our region. The SEER registry does not capture cancer recurrences so we were unable to account for these in our analyses. However, SEER does capture second primary diagnoses, which we included in our analysis (107 people in our analysis had both a prevalent and incident cancer diagnosis). Our results might be less generalizable to more racially and ethnically diverse populations. Finally, we had reduced power for our cancer subtype analyses due to smaller numbers.

Strengths of our study include the use of validated registry-based cancer diagnoses and consensus-based dementia and AD diagnoses using rigorous methods. We follow ACT study members every two years with an in-person clinic or home visit.[26] People with incident cancer diagnoses had the lowest proportion of study withdrawals, making it unlikely that we would have missed dementia or AD diagnoses in people with cancer due to reduced detection or follow-up. In addition, this study took place in a large, population-based sample, making it one of the largest prospective analyses of cancer and dementia/AD to date.

In conclusion, our study results do not support an inverse association between prevalent cancer diagnoses, which were primarily early-stage, less aggressive cancers, and risk of dementia or AD. We did find inverse associations between prevalent cancers diagnosed at late stages with more aggressive tumor biology and dementia and AD risk, and an inverse association incident cancers diagnosed at older ages and AD risk. These results may be reassuring clinically in that there is no increased risk of dementia or AD associated with a prior cancer diagnosis. At the same time, our results among people with late-stage prevalent cancer or incident cancer provide some support to the notion that there may be a biological explanation for the inverse association. Despite biological plausibility, we cannot completely rule out the possibility of bias in our estimates due to issues related to selective mortality. Further investigation as to the biological links between cancer and the risk of neurodegeneration may be warranted. As more people survive cancer in old age in the coming years, we and others may also be able to address longer-term outcomes of cancer treatment in older people, including whether cancer treatment regimens are associated with risk of dementia or AD.

## Supporting information

S1 Table Risks of dementia and AD after a cancer diagnosis among ACT study participants, stratified by cancer stage. This table shows risks of dementia and AD for prevalent and incident cancers stratified by early and late stage cancer diagnoses.
(DOCX)

S2 Table Risks of dementia and AD after a cancer diagnosis among 2,787 ACT study participants who survived to age 80 or older. This table shows risks of dementia and AD for prevalent and incident cancers limited to people who survived to age 80 or older.
(DOCX)

S3 Table Risks of dementia and AD after a breast or prostate cancer diagnosis among ACT study participants. This table shows risks of dementia and AD for prevalent and incident cancers separately for women diagnosed with breast cancer and men diagnosed with prostate cancer.
(DOCX)

S4 Table Risks of Dementia and AD After a Smoking-Related Cancer Diagnosis Among ACT Study Participants. This table shows risks of dementia and AD for prevalent and incident cancers limited to people diagnosed with a smoking-related cancer (oral cavity, pharynx, larynx, esophagus, stomach, pancreas, lung, bladder, or kidney.
(DOCX)
