# Supplementary material for: Risk of Alzheimer's disease or dementia following a cancer diagnosis
Source: PLoS One. 2017 Jun 20;12(6):e0179857. doi: 10.1371/journal.pone.0179857 (PMC5478144; doi:10.1371/journal.pone.0179857)
Supplement: S1 Table — This table shows risks of dementia and AD for prevalent and incident cancers stratified by early and late stage cancer diagnoses. (DOCX) [file pone.0179857.s001.docx]

Supplemental Table 1. Risks of Dementia and AD After a Cancer Diagnosis Among ACT Study Participants, Stratified by Cancer Stage^a^

|  |  | follow-up time  (person-years) | # events | Incidence  per 1000 per year |  | Adjusted HR^b^ |  |
| --- | --- | --- | --- | --- | --- | --- | --- |
|  |  |  |  |  | 95% CI |  | 95% CI |
| **Early stage cancers** | | |  |  |  |  |  |
| **Dementia** |  |  |  |  |  |  |  |
| No cancer |  | 26,735 | 839 | 31.4 | 29.3, 33.6 | 1 |  |
| Prevalent cancer | | 3,852 | 134 | 34.8 | 29.4, 41.2 | 1.02 | 0.83, 1.24 |
| Incident cancer | | 2,098 | 74 | 35.3 | 28.1, 44.3 | 0.84 | 0.64, 1.09 |
| **Possible/Probable AD** | | |  |  |  |  |  |
| No cancer |  | 26,735 | 678 | 25.4 | 23.5, 27.3 | 1 |  |
| Prevalent cancer | | 3,852 | 111 | 28.8 | 23.9, 34.7 | 1.06 | 0.85, 1.32 |
| Incident cancer | | 2,098 | 51 | 24.3 | 18.5, 32.0 | 0.68 | 0.49, 0.94 |
| **Late stage cancers** | | |  |  |  |  |  |
| **Dementia** |  |  |  |  |  |  |  |
| No cancer |  | 26,735 | 839 | 31.4 | 29.3, 33.6 | 1 |  |
| Prevalent cancer | | 925 | 16 | 17.3 | 10.6, 28.2 | 0.51 | 0.30, 0.89 |
| Incident cancer | | 672 | 21 | 31.3 | 10.4, 48.0 | 0.76 | 0.46, 1.26 |
| **Possible/Probable AD** | | |  |  |  |  |  |
| No cancer |  | 26,735 | 678 | 25.4 | 23.5, 27.3 | 1 |  |
| Prevalent cancer | | 925 | 12 | 13.0 | 7.4, 22.8 | 0.50 | 0.27, 0.94 |
| Incident cancer | | 672 | 19 | 28.3 | 18.0, 44.4 | 0.87 | 0.52, 1.46 |

Abbreviations: ACT (Adult Changes in Thought); AD (Alzheimer’s disease); CI (confidence interval); HR (hazard ratio)

^a^Results do not include 41 cancers missing information on stage.

^b^HR uses age as the time scale, and is adjusted for age at ACT study entry, ACT cohort, gender, education, diabetes, hypertension, heart disease, stroke, smoking status, low self-rated health, regular exercise, and BMI
