# Supplementary material for: Risk of Alzheimer's disease or dementia following a cancer diagnosis
Source: PLoS One. 2017 Jun 20;12(6):e0179857. doi: 10.1371/journal.pone.0179857 (PMC5478144; doi:10.1371/journal.pone.0179857)
Supplement: S2 Table — This table shows risks of dementia and AD for prevalent and incident cancers limited to people who survived to age 80 or older. (DOCX) [file pone.0179857.s002.docx]

Supplemental Table 2. Risks of Dementia and AD After a Cancer Diagnosis Among 2,787 ACT Study Participants who Survived to Age 80 or Older

|  |  | follow-up time | # events | Incidence |  | Adjusted HR^a^ |  |
| --- | --- | --- | --- | --- | --- | --- | --- |
| **Dementia** |  | (person-years) |  | (per 1000 per year) | 95% CI |  | 95% CI |
| No cancer |  | 14,548 | 651 | 44.7 | 41.4, 48.3 | 1 |  |
| Prevalent cancer | | 3,125 | 125 | 38.9 | 32.6, 46.3 | 0.92 | 0.74, 1.14 |
| Incident cancer | | 1,715 | 80 | 46.6 | 37.5, 58.1 | 0.78 | 0.60, 1.02 |
| **Possible/Probable AD** | | |  |  |  |  |  |
| No cancer |  | 14,548 | 546 | 37.5 | 34.5, 40.8 | 1 |  |
| Prevalent cancer | | 3,215 | 103 | 32.0 | 26.4, 38.9 | 0.91 | 0.72, 1.15 |
| Incident cancer | | 1,715 | 60 | 35.0 | 27.2, 45.1 | 0.69 | 0.51, 0.92 |

Abbreviations: ACT (Adult Changes in Thought); AD (Alzheimer’s disease); CI (confidence interval); HR (hazard ratio)

^a^HR uses age as the time scale with age 80 as the point of entry, and is adjusted for age at ACT study entry, ACT cohort, gender, education, diabetes, hypertension, heart disease, stroke, smoking status, low self-rated health, regular exercise, and BMI
