# Supplementary material for: Risk of Alzheimer's disease or dementia following a cancer diagnosis
Source: PLoS One. 2017 Jun 20;12(6):e0179857. doi: 10.1371/journal.pone.0179857 (PMC5478144; doi:10.1371/journal.pone.0179857)
Supplement: S3 Table — This table shows risks of dementia and AD for prevalent and incident cancers separately for women diagnosed with breast cancer and men diagnosed with prostate cancer. (DOCX) [file pone.0179857.s003.docx]

Supplemental Table 3. Risks of Dementia and AD After a Breast or Prostate Cancer Diagnosis Among ACT Study Participants

|  |  | follow-up time  (person-years) | # events | Incidence  per 1000 per year |  | Adjusted HR^a^ |  |
| --- | --- | --- | --- | --- | --- | --- | --- |
| **Breast cancer**  **(females only)** | |  |  |  | 95% CI |  | 95% CI |
| **Dementia** |  |  |  |  |  |  |  |
| No cancer^b^ |  | 16,152 | 518 | 32.1 | 29.4, 35.0 | 1 |  |
| Prevalent breast cancer | | 1,398 | 48 | 34.3 | 25.9, 45.6 | 1.09 | 0.78, 1.51 |
| Incident breast cancer | | 786 | 29 | 36.9 | 25.6, 53.1 | 0.82 | 0.53, 1.26 |
| **Possible/Probable AD** | | |  |  |  |  |  |
| No cancer^b^ |  | 16,152 | 432 | 26.7 | 24.3, 29.4 | 1 |  |
| Prevalent breast cancer | | 1,398 | 41 | 29.3 | 21.6, 39.8 | 1.14 | 0.80, 1.62 |
| Incident breast cancer | | 786 | 24 | 30.5 | 20.5, 45.5 | 0.81 | 0.50, 1.29 |
| **Prostate cancer (males only)** | | |  |  |  |  |  |
| **Dementia** |  |  |  |  |  |  |  |
| No cancer^b^ |  | 10,275 | 312 | 30.4 | 27.2, 33.9 | 1 |  |
| Prevalent prostate cancer | | 838 | 23 | 27.5 | 18.2, 41.3 | 0.77 | 0.49, 1.22 |
| Incident prostate cancer | | 692 | 23 | 33.2 | 22.1, 50.0 | 1.05 | 0.66, 1.68 |
| **Possible/Probable AD** | | |  |  |  |  |  |
| No cancer^b^ |  | 10,275 | 237 | 23.1 | 20.3, 26.2 | 1 |  |
| Prevalent prostate cancer | | 838 | 19 | 22.7 | 14.5, 35.6 | 0.80 | 0.48, 1.34 |
| Incident prostate cancer | | 692 | 14 | 20.2 | 12.0, 34.2 | 0.79 | 0.43, 1.43 |

Abbreviations: ACT (Adult Changes in Thought); AD (Alzheimer’s disease); CI (confidence interval); HR (hazard ratio)

^a^HR uses age as the time scale and is adjusted for age at ACT study entry, ACT cohort, education, diabetes, hypertension, heart disease, stroke, smoking status, low self-rated health, regular exercise, and BMI

^b^Reference group with “no cancer” includes all females (for breast cancer analysis) or all males (for prostate cancer analysis) who have never had any type of cancer.
