# Supplementary material for: Risk of Alzheimer's disease or dementia following a cancer diagnosis
Source: PLoS One. 2017 Jun 20;12(6):e0179857. doi: 10.1371/journal.pone.0179857 (PMC5478144; doi:10.1371/journal.pone.0179857)
Supplement: S4 Table — This table shows risks of dementia and AD for prevalent and incident cancers limited to people diagnosed with a smoking-related cancer (oral cavity, pharynx, larynx, esophagus, stomach, pancreas, lung, bladder, or kidney. (DOCX) [file pone.0179857.s004.docx]

Supplemental Table 4. Risks of Dementia and AD After a Smoking-Related^a^ Cancer Diagnosis Among ACT Study Participants

|  |  | follow-up time  (person-years) | # events | Incidence  (per 1000 per year) |  | Adjusted HR^b^ |  |
| --- | --- | --- | --- | --- | --- | --- | --- |
| **Dementia** |  |  |  |  | 95% CI |  | 95% CI |
| No cancer^c^ |  | 26,735 | 839 | 31.4 | 29.3, 33.6 | 1 |  |
| Prevalent smoking-related cancer | | 408 | 14 | 34.3 | 20.3, 58.0 | 0.98 | 0.57, 1.71 |
| Incident smoking-related cancer | | 215 | 7 | 32.5 | 15.5, 68.3 | 0.86 | 0.40, 1.81 |
| **Possible/Probable AD** | | |  |  |  |  |  |
| No cancer^c^ |  | 26,735 | 678 | 25.4 | 23.5, 27.3 | 1 |  |
| Prevalent smoking-related cancer | | 408 | 11 | 27.0 | 14.9, 48.7 | 0.97 | 0.52, 1.82 |
| Incident smoking-related cancer | | 215 | 7 | 32.5 | 15.5, 68.3 | 1.00 | 0.47, 2.11 |

Abbreviations: ACT (Adult Changes in Thought); AD (Alzheimer’s disease); CI (confidence interval); HR (hazard ratio)

^a^Smoking related cancers include oral cavity, pharynx, larynx, esophagus, stomach, pancreas, lung, bladder, or kidney

^b^HR uses age as the time scale and is adjusted for age at ACT study entry, ACT cohort, gender, education, diabetes, hypertension, heart disease, stroke, smoking status, low self-rated health, regular exercise, and BMI

^c^Reference group with “no cancer” includes only people who have never had any type of cancer.
